# Comparative Anatomical Study Between the Human and Swine Liver and Its Importance in Xenotransplantation

**DOI:** 10.7759/cureus.9411

**Published:** 2020-07-27

**Authors:** Athanasios Ntonas, Anastasios Katsourakis, Nikiforos Galanis, Eva Filo, George Noussios

**Affiliations:** 1 Public Health, National School of Public Health, Thessaloniki, GRC; 2 Surgery, General Hospital of Thessaloniki "O Agios Dimitrios", Thessaloniki, GRC; 3 Orthopedics, School of Medicine, Aristotle University of Thessaloniki, Thessaloniki, GRC; 4 Pediatric Surgery, Agios Dimitrios - Georgios Gennimatas, Thessaloniki, GRC; 5 Physical Education and Sports Sciences, Aristotle University of Thessaloniki, Thessaloniki, GRC; 6 Otolaryngology, Aristotle University of Thessaloniki, Thessaloniki, GRC

**Keywords:** liver, pig, human, xenotransplantation, comparative anatomy

## Abstract

The liver is a multifunctional organ; due to its functional and structural complexity, there are many factors that may lead it to function inadequately, a state called liver failure. Transplantation is the only appropriate therapy for patients in cases of liver failure. However, there are many limitations to this treatment, and the scientific community has considered methods based on pigs because of their unique structural and cellular compatibility with humans. In this review, we conducted an anatomic comparative study of the liver’s parenchyma and vascular network between humans and pigs to extract useful information for xenotransplantation and autologous cell or organ generation in pigs. We reviewed articles from 2007 to 2019 and used the databases of Scopus, PubMed and Google Scholar. We concluded that, despite the difference concerning the shape of the human and pig livers, the number of segments and the bile and vascular system are similar, making the pig liver useful in experimental surgery for xenotransplantation.

## Introduction and background

The liver’s functions are complex and fundamental for life. The organ’s parenchymal cells synthesize most of the factors and inhibitors of the clotting and fibrinolytic systems [1]. This organ also plays a major role in metabolism [2]. Furthermore, the liver seems to be a frontline immune organ with functions related to the detection, capture and clearing of bacteria, viruses and macromolecules entering the body via the gut [3]. Due to its functional and structural complexity, there are many factors leading to liver failure [4]. Liver transplantation is the only appropriate therapy for patients with end-stage organ disease [5]. However, due to their limitations, the scientific community considered using methods like xenotransplantation from animals that had been genetically engineered to be less immunogenic [6,7], differentiation of autologous stem cells into hepatocyte-like cells [8], and the autologous organ generation in xenogeneic animals [9]. Many investigations on medical treatment and surgical techniques have been applied to pigs; this is feasible due to their great structural and cellular compatibility with humans, making them perfect candidates for liver xenotransplantation [10]. Inspired by the comparative study between rodent and human livers by Kruepunga et al. in 2019 [11], the purpose of this review is to compare anatomical elements of the liver in between the human and porcine cases to enhance the current knowledge based on the recent literature.

## Review

Information retrieval

We conducted an anatomic comparative study of the liver’s parenchyma and vascular network between humans and pigs to extract useful information for xenotransplantation and autologous cell or organ generation in pigs. The articles qualified for this review were extracted from the databases of Scopus, PubMed and Google Scholar. The search terms that were used were as follows: liver, swine, pig, xenotransplantation, human, transplantation, and liver failure. No review protocol existed. The references of all the included articles were searched to identify if any further relevant articles existed. For the analysis, we included only original articles written in English during the last 12 years. Case reports, conference abstracts, letters to the editor and studies reporting incomplete or irrelevant data were excluded from the study.

The liver structure

**Figure 1:**
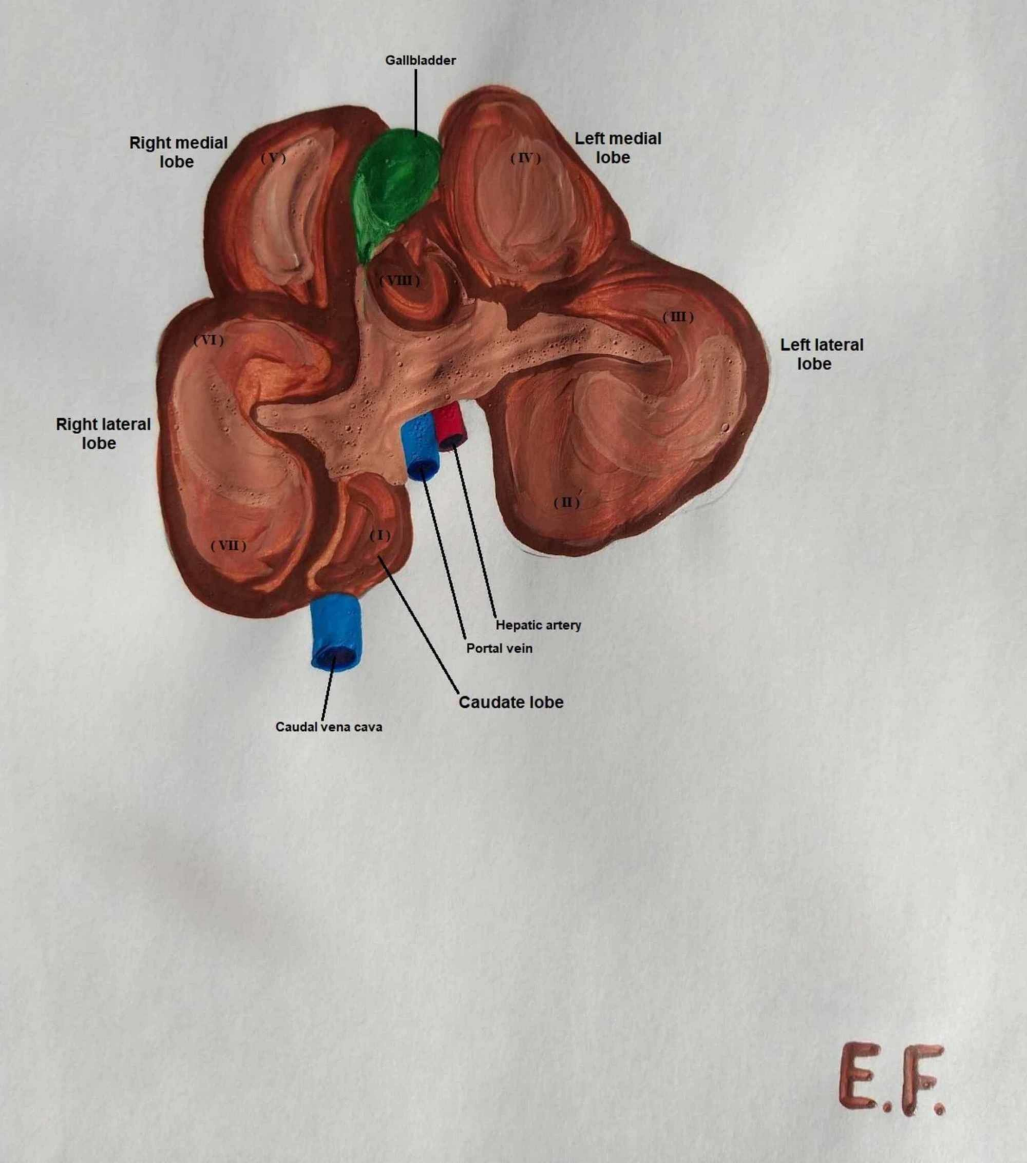
Caudoventral view of the porcine liver, demonstrating the segments

The liver is the body's largest intraperitoneal gland. In swine, the liver is situated on the front right side of the abdominal cavity, behind the diaphragm. The shape of the porcine liver is lobular and thick in the centre, becoming thinner at the perimeter of the organ. It consists of four lobes and eight segments. The sinister hepatic lobe is divided into the lobus hepatis sinister lateralis, with segments II and III, and lobus hepatis sinister medialis, with segment IV; the dexter hepatic lobe is divided into the lobus hepatis dexter lateralis, with segments I, VII and VI, and lobus hepatis dexter medialis, with segments VIII and V. Between the sinister medialis and the dexter medialis hepatis lobi and along the gallbladder, there is the smaller and very thin quadrate lobe. There is also a sixth lobe attached to the dexter lateralis hepatis lobus called the caudate lobe (Figure 1) [12].

**Figure 2:**
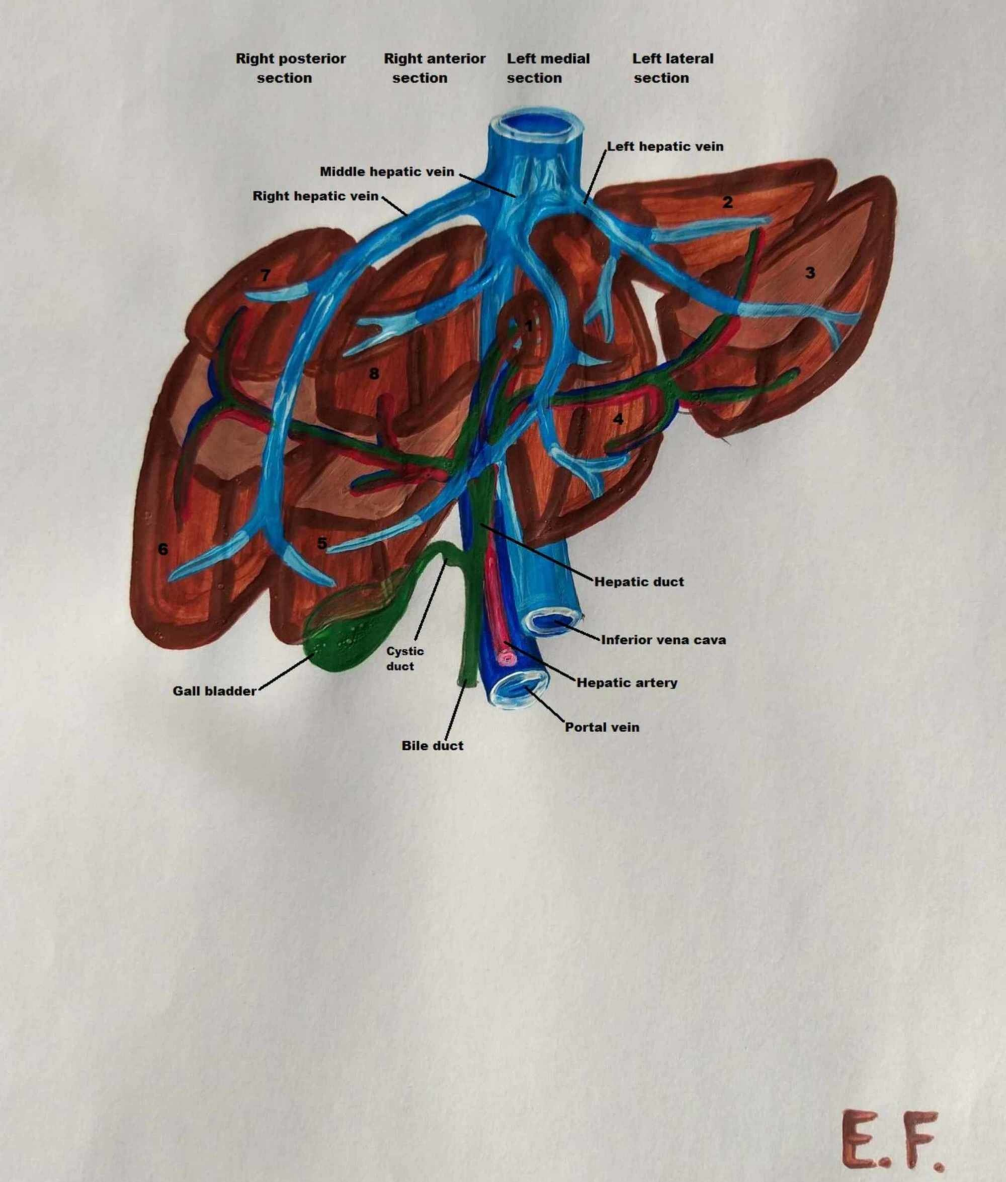
Segments of the human liver, biliary system and vasculature

In the human body, the liver is found in the upper quadrant of the abdominal cavity, below the diaphragm and over the stomach. The human liver has a triangular prism shape, and the size decreases from right to left. According to embryology the liver primordium takes place on Day 22 after conception [13]. Croisille and Le Douarin hypothesize three separate inductive processes acting on the endoderm. First, we have the formation of the hepatocardiac mesoderm which as the hepatic bud appears it splits to the hepatic and cardiac mesenchyme. After the second and third induction, the cells of the endodermal cords differentiate into hepatocytes and we have the formation of the liver sinusoids. By Day 51 the intrahepatic veins achieve the normal segmentation and distribution, while the arteries and the bile duct do not progress so quick to their adult form [14]. Anatomically, it is divided into the left, right, caudate and quadrate lobes [15]. The liver is also divided into eight segments with an autonomous vascular network (Figure 2).

The hepatic artery

The human liver is supplied with blood from two main vessels - the portal vein and the hepatic artery. While the hepatic veins and their biliary system transfer blood into the cardiac circulation, the hepatic artery consists of two branches, the left and right hepatic artery, which are linked to each segment of the liver (Figure 2) [16].

**Figure 3:**
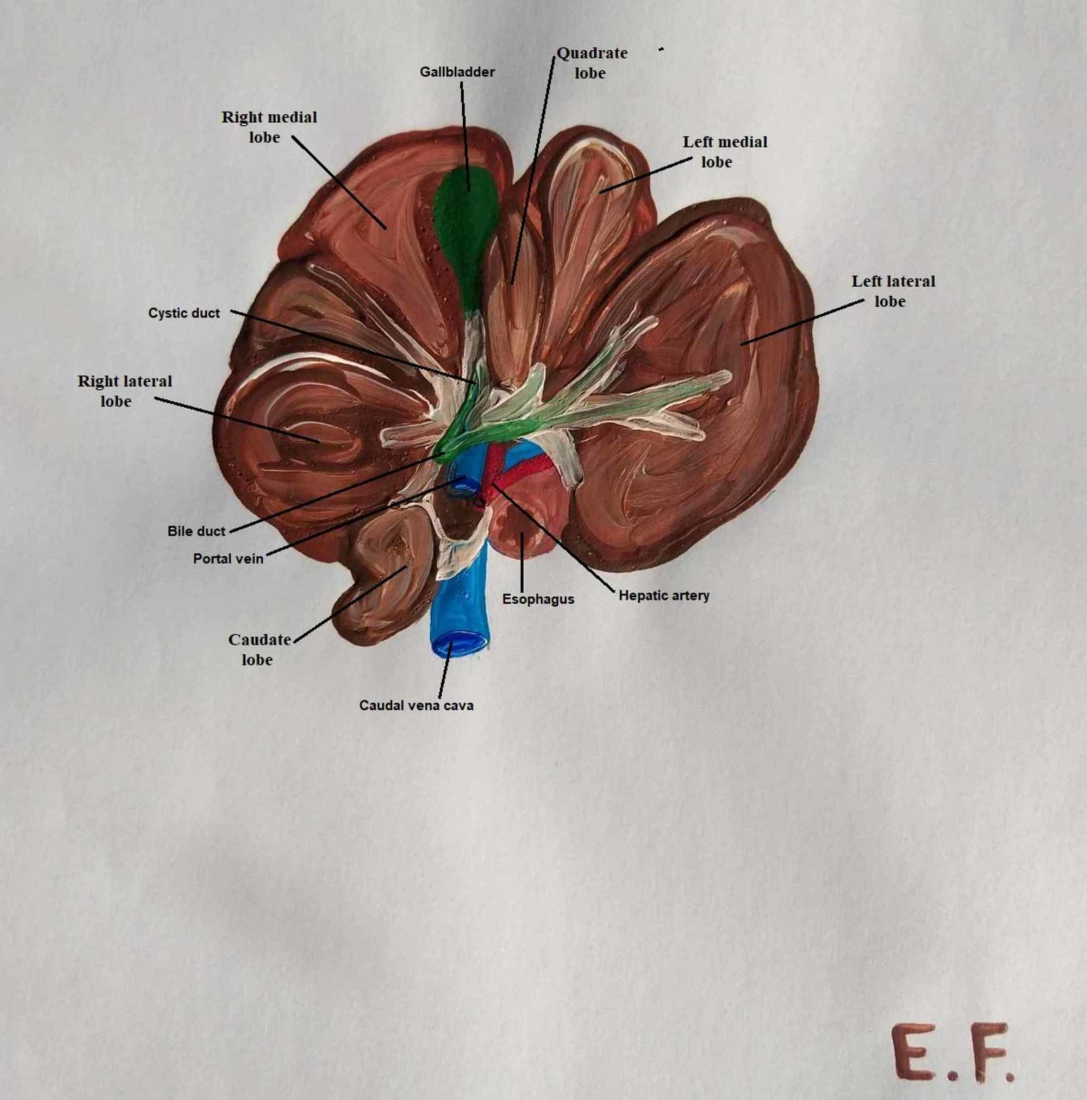
Pig liver anatomy: lobes, biliary system and vessels

In swine, the left gastric, splenic and hepatic arteries form the basin celiac artery, which is a branch of the abdominal aorta. The hepatic artery in pigs is divided into left and right branches. The left branch is divided into the ramus sinister medialis and ramus sinister lateralis, the main arterial vasculature of the left lobe, and the ramus quadratus, which supplies the corresponding lobe and gallbladder. The right branch is also separated into two branches, the ramus dexter medialis and ramus dexter lateralis, which supply the right lobe of the liver (Figure 3) [12].

The portal vein

The portal vein delivers blood from the gastrointestinal tract, pancreas and spleen to the liver. In pigs, the portal vein is formed ventrally out of the posterior vena cava and behind the pancreatic gland, from the superior and anterior mesenteric vein and splenic vein. The vein is divided into left and right branches at the hilum. The left ramus is longer and wider than the right ramus and corresponds to the segments of the left median and left lateral lobes. The right ramus is bisected into the right lateral and right median branches. The right lateral branch corresponds to segments I, VI and VII, while the median branch corresponds to segments V and VIII. It is significant that there are communicating branches of the portal vein between the right median and right lateral rami (Figure 3) [12].

In the human body, the splenic and superior mesenteric veins are merged behind the neck of the pancreas to form the portal vein. The portal vein is divided into the sinister and dexter branches before entering the liver parenchyma. The dexter portal vein gives off posterior branches, providing blood to segments VI and VII, as well as anterior branches leading to segments V and VIII of the dexter hepatic lobe. The longer sinister portal vein is divided into four branches, the first two corresponding to segment IV and the other two to segments II and III (Figure 2) [17].

Hepatic veins

In swine, liver drainage occurs via four main veins. The dexter lateral and dexter medial veins drain segments of lobus dexter medialis and dexter lateralis, except segment I, which drains straight into the vena cava. The sinister lateral vein pumps blood from segments III and II of the lobus sinister lateralis, while the sinister lateral ramus drains blood from segment IV of the left medial lobe (Figure 3) [12].

In humans, the venous drainage of the liver is accomplished by the right, left and middle hepatic veins, which meet the vena cava. The left hepatic vein is composed of two branches, and it drains the left lobe and segment IV of the right lobe. The right hepatic vein is larger than the left and middle veins; it comprises four smaller branches and drains segments V, VI, VII and VIII of the right lobe (Figure 2) [18].

The biliary system

According to anatomical data, the common bile duct is the primary biliary canal of the liver. In the human liver, the common bile duct is divided into the cystic duct and common hepatic duct. The first leads to the gallbladder; the latter splits into left and right hepatic ducts before leaving the liver parenchyma. The hepatic ducts, hepatic arteries and portal vein form the Glissonian sheaths; they have a common intrahepatic course and drain the same segments as the portal vein. Therefore, the left hepatic duct drains bile from segments I, II, III, and IV, while the right hepatic duct drains bile from segments V, VI, VII and VIII (Figure 2) [18, 19].

In pigs, the ductuli interlobulares, which are formed of small bile ducts, make their intralobular course in the parenchyma with the portal vein in the Glissonian sheaths. The ductuli interlobulares sum up to the dexter and sinister hepatic duct. The right hepatic duct drains the right medial, right lateral and caudate lobes, while the left hepatic bile duct drains the left medial, left lateral and quadrate lobes. From the confluence of the sinister and dexter hepatic duct is formed the common hepatic bile duct, which is anastomosed near the porta hepatis with the ductus cysticus to form the common bile duct. The cystic duct leads from the neck of the gallbladder to the common bile duct. The bile segmentation in pigs is also the same as that of the portal vein due to their common track in the liver parenchyma. Therefore, the left hepatic duct drains bile from segments II, III and IV, while the right hepatic duct drains bile from segments I, V, VI, VII and VIII (Figure 3) [12].

Discussion

According to our literature review, the human liver consists of four lobes. In the diaphragmatic surface, we can observe only left and right lobe separated by the falciform ligament [20]. From the visceral surface, we can observe the caudate and quadrate lobe. The caudate lobe is posterior to the porta hepatis, anterior to the inferior vena cava and lateral to the ligamentum venosum, while the quadrate lobe is demarcated medially by the round ligament of the liver and posteriorly by the porta hepatis. The gallbladder is situated in the visceral surface of the liver sidelong to the quadrate lobe [15]. Segments I, II and III form the left liver lobe, while segments IV, V, VI, VII and VIII form the right liver lobe (Figure 2) [11]. Both human and pig livers consist of sinister, dexter, quadrate and caudate lobes; because of the more lobular shape of the porcine liver, the sinister and dexter lobes are each divided into lateral and medial lobes.

The liver’s vascular system in humans consists of the hepatic artery, portal vein and hepatic vein. Blood flows from the abdominal aorta to a branch called the celiac artery. This artery is divided into the left gastric, splenic and hepatic arteries [21]. The hepatic artery delivers 25% of the incoming blood to the liver. The remaining 75% is supplied from the portal venous system, which is formed from the splenic vein and superior mesenteric vein [17]. In both humans and pigs, the arterial vascular system is identical. The portal veins of the two species have minor differences. In humans, the division of the portal vein occurs outside the liver parenchyma, while in swine, it occurs at the hilum. In the pig liver, there are also communicating branches between the right median and right lateral rami of the portal vein [12]. According to the literature, in humans, the medial hepatic vein shares the drainage of segment IV with the left hepatic vein and the drainage of segments V and VIII with the right hepatic vein. However, in some cases, the middle and left hepatic veins have a common entrance to the vena cava. In swine, the dexter lateral and medial branches are united separately with the vena cava, whereas sometimes, like in the human liver anatomy, the left medial vein merges with the left lateral vein into a common stem before the anastomosis with the vena cava [12]. Despite the similarities of human and pig venous drainage systems, the pig liver’s posterior vena cava passes through the liver parenchyma and drains segment I, which increases the difficulty in partial hepatectomy operations.

The liver’s biliary system is the primary network for metabolic products to be removed from the body via bile. The human and pig biliary systems are anatomically and functionally similar. The only difference between them is the drainage of segment I, which is done by the sinister hepatic duct in humans, while in swine, it is drained by the dexter hepatic duct.

Liver transplantation is the only effective treatment for liver failure. However, the treatment’s success relies on the number of available donors, and since donors are lacking, thousands of patients are dying while still on the waiting list. Another barrier is the amount of time a patient needs for immunosuppression to try to ensure that his or her body will accept the transplant. To reduce mortality stemming from the length of the waiting list, the transplant of organs from donors after cardiac death can be employed as there are no significant differences in patient and graft survival between those of donors after cardiac death and those after brain death [22]. A more promising solution is using genetically engineered xenogeneic species for xenotransplantation. With genetically engineered animals, the remaining antibody response is reduced by deleting the responsible genes from the animal’s genome [6].

## Conclusions

In conclusion, there are many studies on medical treatment and surgical techniques applied in pigs. This is feasible due to the animal's great structural and cellular compatibility with humans. Despite the small differences discussed in this review, the pig is a perfect candidate for liver xenotransplantation.

Due to recent medical progress, liver xenotransplantation from a genetically engineered pig donor increases recipient survival up to nine days postoperatively, with adequate liver function. Given the medicine evolution of the last decades, in the near future, xenotransplantation may be the solution for patients with liver failure.
